# Individual differences in interoceptive accuracy and prediction error in motor functional neurological disorders: A DTI study

**DOI:** 10.1002/hbm.25304

**Published:** 2020-12-07

**Authors:** Petr Sojka, Ibai Diez, Martin Bareš, David L. Perez

**Affiliations:** ^1^ Department of Psychiatry, Faculty of Medicine Masaryk University Brno and University Hospital, Brno Brno Czech Republic; ^2^ Department of Psychology and Psychosomatics, Faculty of Medicine Masaryk University and University Hospital Brno Brno Czech Republic; ^3^ Department of Neurology, Functional Neurological Disorder Research Program, Behavioral Neurology Unit, Massachusetts General Hospital Harvard Medical School Boston Massachusetts USA; ^4^ Athinoula A. Martinos Center for Biomedical Imaging, Massachusetts General Hospital Harvard Medical School Charlestown Massachusetts USA; ^5^ Gordon Center, Department of Nuclear Medicine, Massachusetts General Hospital Harvard Medical School Boston Massachusetts USA; ^6^ First Department of Neurology, Faculty of Medicine Masaryk University and St. Anne's University Hospital Brno Czech Republic; ^7^ Department of Neurology, School of Medicine University of Minnesota Minneapolis Minnesota USA; ^8^ Department of Psychiatry, Neuropsychiatry Unit, Massachusetts General Hospital Harvard Medical School Boston Massachusetts USA

**Keywords:** conversion disorder, diffusion tensor imaging, dissociative seizures, DTI, functional movement disorder, interoception, psychogenic

## Abstract

In motor functional neurological disorders (mFND), relationships between interoception (a construct of high theoretical relevance to its pathophysiology) and neuroanatomy have not been previously investigated. This study characterized white matter in mFND patients compared to healthy controls (HCs), and investigated associations between fiber bundle integrity and cardiac interoception. Voxel‐based analysis and tractography quantified fractional anisotropy (FA) in 38 mFND patients compared to 38 HCs. Secondary analyses compared functional seizures (FND‐seiz; *n* = 21) or functional movement disorders (*n* = 17) to HCs. Network lesion mapping identified gray matter origins of implicated fiber bundles. Within‐group mFND analyses investigated relationships between FA, heartbeat tracking accuracy and interoceptive trait prediction error (discrepancies between interoceptive accuracy and self‐reported bodily awareness). Results were corrected for multiple comparisons, and all findings were adjusted for depression and trait anxiety. mFND and HCs did not show any between‐group interoceptive accuracy or FA differences. However, the FND‐seiz subgroup compared to HCs showed decreased integrity in right‐lateralized tracts: extreme capsule/inferior fronto‐occipital fasciculus, arcuate fasciculus, inferior longitudinal fasciculus, and thalamic/striatum to occipital cortex projections. These alterations originated predominantly from the right temporoparietal junction and inferior temporal gyrus. In mFND patients, individual differences in interoceptive accuracy and interoceptive trait prediction error correlated with fiber bundle integrity originating from the insula, temporoparietal junction, putamen and thalamus among other regions. In this first study investigating brain‐interoception relationships in mFND, individual differences in interoceptive accuracy and trait prediction error mapped onto multimodal integration‐related fiber bundles. Right‐lateralized limbic and associative tract disruptions distinguished FND‐seiz from HCs.

## Graphical abstract

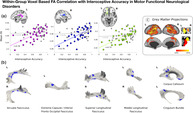

## 1 INTRODUCTION

Functional neurological disorder (FND) is a common neuropsychiatric condition resulting in substantial disability and healthcare costs (Espay, Aybek, et al., 2018; Gelauff, Carson, Ludwig, Tijssen, & Stone, 2019; Stephen, Fung, Lungu, & Espay, 2020). After more than 100 years in the medical literature, the pathophysiology of FND remains incompletely understood (Baizabal‐Carvallo, Hallett, & Jankovic, 2019; Bègue, Adams, Stone, & Perez, 2019). Experimental paradigms and neuroimaging methods, together with conceptual models (Edwards, Adams, Brown, Pareés, & Friston, 2012; Keynejad et al., 2019; Pick, Goldstein, Perez, & Nicholson, 2018; Sojka, Bareš, Kašpárek, & Světlák, 2018), can now interrogate neural circuits in FND related to interoception, self‐agency, emotion processing, and attentional biases among other candidate constructs (Drane et al., 2020). Interoception, the construct of interest for this study, is the process of detecting, interpreting, and integrating internal bodily signals (Khalsa et al., 2018; Paulus, Feinstein, & Khalsa, 2019). Misattribution of bodily sensations may contribute to altered bodily and emotional awareness in the pathophysiology of FND (Perez et al., 2015). Additionally, while functional magnetic resonance imaging (fMRI) has been used by several groups to elucidate the neurobiology of FND (Aybek et al., 2014; Diez et al., 2020; Espay, Maloney, et al., 2018; Wegrzyk et al., 2018), minimal attention has been given to white matter anatomy and relationships between fiber bundle integrity and interoceptive accuracy. These gaps, if clarified, can provide important mechanistic insights into the pathophysiology of FND.

Across the spectrum of motor FND (mFND), which we frame as encompassing functional [psychogenic nonepileptic/dissociative] seizures (FND‐seiz) and functional movement disorders (FND‐movt), distributed corticolimbic abnormalities spanning multiple brain networks have been identified (Baizabal‐Carvallo et al., 2019; Perez et al., 2015; Pick, Goldstein, et al., 2018; Szaflarski & LaFrance, 2018). Findings include heightened amygdala reactivity to affective stimuli (Aybek et al., 2015; Aybek, Nicholson, Zelaya, et al., 2014; Voon, Brezing, et al., 2010), increased limbic‐motor control network connectivity (Aybek et al., 2015; Aybek, Nicholson, Zelaya, et al., 2014; Diez, Ortiz‐Terán, et al., 2019; van der Kruijs et al., 2012; Voon, Brezing, et al., 2010), altered right temporoparietal junction (TPJ) activity and connectivity (Arthuis, Micoulaud‐Franchi, Bartolomei, McGonigal, & Guedj, 2014; Baek et al., 2017; Maurer et al., 2016; Voon, Gallea, et al., 2010), and default‐mode network abnormalities among other findings (Monsa, Peer, & Arzy, 2018). Structural MRI studies in mFND compared to healthy controls (HCs) also report increased amygdala gray matter volume (Maurer et al., 2018), cingulo‐insular atrophy (Labate et al., 2012; Perez, Williams, et al., 2017), and changes in sensorimotor and striatothalamic areas (Aybek et al., 2014; Bègue et al., 2019; Espay, Maloney, et al., 2018). Several of these functional and structural (gray matter) findings overlap with the salience network, comprised of cingulo‐insular, amygdalar and periaqueductal gray (PAG) areas, and implicated in interoception, homeostasis, and emotional awareness (Khalsa et al., 2018; Kleckner et al., 2017; Seeley, 2019). Additionally, cingulo‐insular areas, together with the TPJ, are involved in multimodal integration—which helps contextualize how these brain areas may relate to fundamental disturbances in self‐agency and emotional awareness found in mFND (Baek et al., 2017; Diez, Ortiz‐Terán, et al., 2019; Sepulcre, Sabuncu, Yeo, Liu, & Johnson, 2012; Voon, Gallea, et al., 2010). FND‐seiz and FND‐movt cohorts also share clinical features and predisposing vulnerabilities (Gray, Calderbank, Adewusi, Hughes, & Reuber, 2020; McKenzie, Oto, Graham, & Duncan, 2011), yet the majority of research in mFND to date has been performed in discrete subgroups. Thus, a transdiagnostic approach is needed to characterize common and subtype‐specific neural circuit components.

Compared to other neuroimaging modalities, white matter research in mFND is in its early stages. By measuring the movement of water molecules in vivo, diffusion tensor imaging (DTI) characterizes white matter microstructural integrity. Fractional anisotropy (FA) is a global microstructural integrity measure, with reduced FA linked to decreased white matter integrity. DTI studies found abnormal integrity in the uncinate fasciculus, corona radiata and internal/external capsule fiber bundles in two small FND‐seiz samples (Hernando, Szaflarski, Ver Hoef, Lee, & Allendorfer, 2015; Lee et al., 2015). Compared to traumatic brain injury controls, patients with FND‐seiz and traumatic brain injury showed decreased integrity in the cingulum bundle, uncinate fasciculus and stria terminalis/fornix (Goodman et al., 2020). Using a graph theory approach, an FND‐seiz cohort showed altered sensorimotor, attentional, and default‐mode network white matter connectivity (Ding et al., 2013). White matter abnormalities were also identified in the dystonia subtype of FND‐movt (*n* = 44); compared to HCs, patients with functional dystonia exhibited decreased integrity in major long‐range associative tracts, the splenium of the corpus callosum, corticospinal tract, and brainstem among other findings (Tomic et al., 2018). Another study in 32 patients with mFND also found reduced white matter integrity in several limbic and associative fiber bundles compared to HCs (Diez, Williams, Kubicki, Makris, & Perez, 2019).

In support of a role for interoception in the pathophysiology of mFND, studies have identified discrepancies between objectively measured physiological reactions and subjective emotional responses (Apazoglou, Mazzola, Wegrzyk, Polara, & Aybek, 2017; Pick, Mellers, & Goldstein, 2018; Roberts et al., 2012; Seignourel et al., 2007), suggesting a failure to accurately integrate or interpret viscerosensory information. For example, one study identified a higher subjective response to social stress in mFND patients, while concurrently showing normal cortisol responses (Apazoglou et al., 2017). Mismatches between subjective and objective symptom reports in FND‐movt patients have also been described (Pareés et al., 2012). Using a heartbeat detection task, interoceptive accuracy deficits have been reported in both FND‐seiz (Koreki et al., 2020) and FND‐movt (Ricciardi, Demartini, Crucianelli, Edwards, & Fotopoulou, 2014) cohorts, although a lack of group‐level differences has also been characterized in other studies (Jungilligens et al., 2019; Pick et al., 2020). In a study by Koreki and colleagues, discrepancies between interoceptive accuracy and self‐reported bodily awareness (an index of interoceptive trait prediction error [ITPE]) correlated with dissociation severity, underscoring the relevance of this measure (Koreki et al., 2020). Here, we aimed to characterize the white matter neuroanatomy of mFND and investigate how individual differences in white matter integrity related to cardiac interoceptive accuracy and ITPE.

Diffusion‐weighted analyses were first performed to examine white matter integrity in 38 mFND patients (FND‐seiz, *n* = 21; FND‐movt, *n* = 17) and 38 HCs. Using a transdiagnostic approach, voxel‐based analyses (VBA) coupled with probabilistic tractography quantified FA differences in all mFND patients compared to HCs. Secondary analyses separately evaluated white matter integrity in FND‐seiz or FND‐movt subgroups compared to HCs. Network lesion mapping analyses identified gray matter origins of implicated fiber bundles. Within‐group relationships between fiber bundle integrity, heartbeat detection performance and ITPE were investigated using VBA, tractography and network lesion mapping. Our hypotheses were several fold: (a) patients with mFND would exhibit worse cardiac interoceptive accuracy compared to HCs; (b) mFND patients would demonstrate reduced integrity in cingulo‐insular (extreme capsule, arcuate fasciculus, cingulum bundle) and amygdala (stria terminalis/fornix) related white matter tracts compared to HCs, with FND‐seiz and FND‐movt showing overlapping profiles; (c) given the salience network's role in the neurobiology of mFND and interoception, we hypothesized that individual differences in interoceptive accuracy and ITPE would correlate with salience network white matter profiles across patients with mFND.

## 2 MATERIALS AND METHODS

### 2.1 Participants and questionnaires

All participants provided written informed consent and the study was approved by the Masaryk University and St. Anne's Hospital ethics committees. Thirty‐eight adults with clinically‐established mFND (32 women, 6 men; age = 34.7 ± 13.2; average illness duration = 4.3 ± 2.8), diagnosed using “rule‐in” clinical criteria (American Psychiatric Association, 2013), were recruited consecutively from the Masaryk University Neurology Clinic between April 2016 and June 2019. All individuals had symptoms for over 2 years. The cohort consisted of video‐electroencephalography documented FND‐seiz (*n* = 21; 19 women, 2 men; age = 23.0 ± 2.8; *n* = 6, major motor; *n* = 8, minor motor; *n* = 7, atonic [Groppel, Kapitany, & Baumgartner, 2000]) and clinically‐established FND‐movt (*n* = 17; 12 women, 5 men; age = 44.0 ± 12.7) patients. Exclusion criteria for all patients included age < 18 years old, MRI abnormality, intellectual disability, major neurological/medical conditions (e.g., epilepsy), psychotic spectrum disorders, bipolar disorder and substance use disorder. Thirty‐eight HCs (32 women, 6 men; age = 34.8 ± 14.1) with no known medical, neurological or psychiatric conditions were recruited from the community through local advertisements. See Table 1 for additional demographic and clinical information in the mFND cohort.

**TABLE 1:** Demographic characteristics of patients with motor functional neurological disorders

Group	Sex	Age	Educ (y)	mFND symptoms	SSRI/SNRI	Other medication
FND‐movt	F	53	13	Dystonia	Citalopram	—
FND‐movt	F	47	11	Parkinsonism	Sertraline	—
FND‐movt	F	57	11	Right hand tremor	—	—
FND‐movt	M	21	10	Gait difficulties	—	—
FND‐movt	F	30	11	Left hand tremor	—	—
FND‐movt	F	60	18	Bilateral leg weakness	—	—
FND‐movt	F	64	13	Gait difficulties	—	—
FND‐movt	M	62	13	Myoclonus	—	—
FND‐movt	F	52	13	Right hand tremor	—	—
FND‐movt	F	32	18	Bilateral arm tremors	—	—
FND‐movt	M	21	9	Myoclonus	—	Clonazepam
FND‐movt	F	30	12	Bilateral leg weakness and speech difficulties	—	—
FND‐movt	F	42	11	Right leg tremor	Citalopram	—
FND‐movt	M	25	13	Dystonia	—	—
FND‐movt	F	22	11	Bilateral arm weakness and tremors	—	—
FND‐movt	F	38	11	Right hand tremor and bilateral leg weakness	Sertraline	—
FND‐movt	F	35	9	Right hand tremor	Venlafaxine	—
FND‐seiz	F	21	10	Major motor	—	Pregabalin
FND‐seiz	F	22	10	Major motor	—	—
FND‐seiz	F	22	13	Minor motor	—	—
FND‐seiz	M	42	10	Major motor	Venlafaxine	—
FND‐seiz	F	28	13	Minor motor	—	—
FND‐seiz	F	47	13	Atonic	—	—
FND‐seiz	F	26	18	Atonic	—	—
FND‐seiz	F	21	13	Minor motor	—	Levetiracetam
FND‐seiz	F	20	13	Atonic	—	—
FND‐seiz	F	21	10	Major motor	—	—
FND‐seiz	F	26	10	Minor motor	Citalopram	Clonazepam
FND‐seiz	F	37	13	Atonic	—	—
FND‐seiz	F	20	11	Minor motor	—	—
FND‐seiz	M	48	11	Atonic	Paroxetine	—
FND‐seiz	F	36	12	Minor motor	—	—
FND‐seiz	F	30	16	Major motor	—	—
FND‐seiz	F	38	9	Minor motor	—	—
FND‐seiz	F	29	10	Atonic	—	—
FND‐seiz	F	48	11	Major motor	Citalopram	Pregabalin
FND‐seiz	F	34	11	Minor motor	Fluoxetine	—
FND‐seiz	F	25	12	Atonic	Sertraline	—

*Note*: mFND indicates motor functional neurological disorder; FND‐movt indicates functional neurological disorder with abnormal movements; FND‐seiz, functional neurological disorder with seizures; F, female; M, male; y, years.

Participants completed the Beck Depression Inventory‐II (BDI), Spielberger State–Trait Anxiety Inventory (STAI) and the Body Perception Questionnaire (BPQ) prior to scanning. The BPQ is a self‐report measure of body awareness and autonomic reactivity (Porges, 1993). The BPQ‐awareness 26‐item subscale (5‐point Likert scoring) has been widely used in interoception research (e.g., Bernátová & Světlák, 2017; Critchley, Wiens, Rotshtein, Ohman, & Dolan, 2004), and provides a trait measure of subjective (perceived) sensitivity to internal sensations with excellent internal consistency (Cabrera et al., 2018).

### 2.2 Heartbeat tracking task

Interoceptive abilities were assessed with a validated heartbeat tracking task (HTT) (Schandry, 1981). During this in‐scanner task, participants' heartbeats were monitored via an electrocardiogram (ECG) monitor (BrainVision BrainAmp MR) with three electrodes attached to the chest. Participants were asked to count their heartbeats during 12 random time windows of varying length (15, 18, 21, 24, 27 and 30 s) and after each trial to report the number of heartbeats counted; these time windows were used to optimize the HTT paradigm for fMRI use. Participants were instructed to count only the heartbeats they felt and asked to not estimate heartbeat counts. To ensure basic familiarity with the task prior to in‐scanner performance, all participants first practiced three rounds of heartbeat tracking outside the scanner. In experimental trials, participants were asked to start counting heartbeats when a heart pictogram appeared on a screen and to stop counting when a response window appeared. Responses were made on a 2‐button box, and the task was presented with E‐Prime (Psychology Software Tools Inc, Pittsburgh) during fMRI recording (fMRI results will be reported elsewhere).

For analyses, R‐peaks were extracted from the ECG in BrainVision Analyzer 2.1. (Brain Products) after MRI artifact correction and applying an IIR Butterworth low‐pass filter. Semi‐automated procedures were implemented, with R‐peaks first automatically labeled in BrainVision analyzer and subsequently inspected and manually interpolated by graduate students blinded to group identity. As a final inspection of the raw data, author P.S. visually inspected all tracings to ensure accuracy of R‐peak selections—which were readily apparent across all subjects with usable data. Due to noise and artifact levels, ECG data from 5 mFND patients and 4 HCs were removed. Thus, for the HTT analyses, 33 mFND patients (age = 34.7 ± 13.2; M = 6; F = 27; FND‐seiz = 18; FND‐movt = 15; Body Mass Index (BMI) = 24.3 ± 5.4) and 34 HCs (age = 34.8 ± 14.1; M = 6; F = 28; BMI = 23.5 ± 4.0) were used. The HTT accuracy scores were calculated for each subject as a difference between reported and counted heartbeats:

$$\mathrm{HTT}=\frac{1}{12}\sum 1-\frac{\left|\mathrm{rec}-\text{count}\right|}{\mathrm{rec}}$$

Higher HTT scores indicate better task performance (interoceptive accuracy). To calculate the ITPE score, which represents the degree of discordance between HTT accuracy and BPQ‐awareness subscale scores, HTT accuracy and BPQ‐awareness scores were converted to z‐scores. At the individual‐patient level, ITPE values were calculated as the difference between BPQ‐awareness subscale and HTT accuracy scores. Larger (more positive) ITPE values suggest greater interoceptive prediction error (increased tendencies to overestimate interoceptive abilities).

### 2.3 Statistical analyses of behavioral data

Independent *t* tests and one‐way analysis of variance (ANOVA) were used to calculate group differences in BPQ, BDI and STAI‐trait scores. Analysis of covariance was used to control for the effect of BMI on group differences in cardiac interoceptive accuracy and ITPE. Where the Levene test for equality of variances was violated, df, *t*‐values, *F*‐values and significance values were adjusted using the Welch *t* test or Welch ANOVA. False discovery rate corrected significance values for multiple statistical testing concerns. All reported significance levels are two‐tailed.

### 2.4 Scan acquisition and preprocessing

See Supplementary Methods for a description of T1‐weighted magnetization prepared rapid gradient‐echo (MPRAGE), fluid‐attenuated inversion recovery (FLAIR), and diffusion‐weighted sequences. Preprocessing procedures are also detailed in the Supplementary Methods.

### 2.5 Voxel‐based analysis

VBA was used to characterize white matter fractional anisotropy (FA) in mFND patients for between‐group and within‐group analyses using validated methods (Schwarz et al., 2014). First, the FA maps of each subject were transformed to MNI152 standard space. To remove inter‐subject variability, the FA maps were smoothed with an isotropic Gaussian kernel (sigma of 1 mm). Thereafter, in all analyses a voxel‐wise general linear model (GLM) was applied to white matter voxels adjusting for age, gender, head motion, BDI and STAI‐trait scores. A 2‐class GLM evaluated differences between the complete mFND cohort and HCs as the primary analysis. Secondary between‐group analyses were performed as follows: FND‐seiz vs. HCs; FND‐movt vs. HCs; and FND‐seiz vs. FND‐movt. For within‐group mFND analyses, 1‐class GLMs evaluated associations between FA maps, HTT performance, and ITPE scores; all within‐group analyses were adjusted for age, gender, BMI, head motion, BDI, and STAI‐trait scores (to account for non‐specific mood and anxiety associations with interoception [Eggart, Lange, Binser, Queri, & Müller‐Oerlinghausen, 2019]). For all VBA analyses, a Monte Carlo simulation cluster‐wise correction was used with 10,000 iterations to estimate the probability of false positive clusters with a *p*‐value < .05.

### 2.6 Probabilistic tractography

To identify the fiber bundles implicated by the VBA‐defined white matter blobs for between‐group and within‐group findings, query language informed tractography was used (Diez, Williams, et al., 2019; Wassermann et al., 2016). This approach incorporates information from the starting and endpoints of each fiber bundle as well as brain areas that the tract passes through (i.e., way points).

To use query language definitions, the transformation between diffusion and T1‐weighted images was used to project *FreeSurfer* parcellations and segmentations onto the diffusion space. Then the FSL BEDPOSTX tool was used, with default parameters, to model crossing fibers. Probabilistic tractography was subsequently performed using the FSL PROBTRACKX tool. The query language defined white matter tracts were computed for all HCs. Subsequently, a nonlinear transformation was applied to the tracts to project them to MNI152 space using MRtrix3 software. Tracts from HCs were combined into group‐based fiber bundles. Thereafter, to account for false positives we eliminated fibers with low probability (<0.1%) and high curvature (>70%) based on previously published methods (Diez, Williams, et al., 2019). To remove crossing fibers, we computed a histogram for each voxel with the probability of the passing fibers going through each possible direction. Fibers with a probability lower than 5% of going within ±15° of the highest probability direction were removed. Additionally, fibers passing through voxels with a probability lower than 0.1% were removed. Due to crossing fibers, each white matter voxel can potentially belong to more than one fiber bundle. Using a probabilistic white matter atlas based on the healthy cohort data, we only projected the VBA results in those fiber bundles with at least a 25% probability of mapping onto that specific fiber bundle.

To investigate between‐group FA fiber bundle differences, a 2‐class GLM was used. All between‐group analyses controlled for age, gender, head motion, BDI and STAI‐trait. The primary analysis compared the entire mFND cohort to HCs; secondary analyses separately compared the FND‐seiz and FND‐movt cohorts to HCs, as well as comparing the FND‐seiz and FND‐movt cohorts to one another. All findings were corrected for multiple comparisons using Monte Carlo simulation cluster‐wise correction with 10,000 iterations and a *p*‐value < .05, applied to a whole‐brain white matter mask.

To investigate within‐group associations between fiber bundle integrity, HTT performance, and ITPE scores, 1‐class GLMs adjusting for age, gender, BMI, head motion, BDI, and STAI‐trait were performed. For statistically significant findings, post‐hoc analyses adjusted within‐group findings for: (a) SSRI and/or SNRI use (yes/no); (b) mFND subtype. All findings were corrected for multiple comparisons using Monte Carlo simulation cluster‐wise correction with 10,000 iterations and a *p*‐value < .05, applied to a whole‐brain white matter mask.

### 2.7 Exploratory network lesion mapping

Tractography was also used to identify the gray matter origins of the VBA implicated white matter results based on structural connectivity patterns; this is a form of white matter‐to‐gray matter network lesion mapping (Fox, 2018). A Human Connectome Project cortical parcellation relying on high quality multimodal MRI features (Glasser et al., 2016) and *FreeSurfer* subcortical regions was used to map the VBA blobs to gray matter areas. After transforming both VBA blob and brain parcellation data to individual subject diffusion space, FSL PROBTRACKX tool was used to track 100 fibers from each atlas region in the brain. To account for false positives, we eliminated fibers with low probability (<1%). For each brain parcellation region, we computed the percentage of voxels of the VBA blobs that passed through the fiber tracts starting in a given parcellation region‐of‐interest (ROI). Finally, the mean of all percentages were computed for each ROI (identifying grey matter areas that contributed white matter projections passing through the VBA blob). Values with an overlapping percentage > 10% were displayed.

## 3 RESULTS

### 3.1 Behavioral findings

In the complete cohort, depression (*p* = .006) and trait anxiety (*p* = .0001) scores were elevated in patients with mFND (STAI‐trait = 39.0 ± 9.6, BDI = 20.1 ± 11.5) compared to HCs (STAI‐trait = 32.9 ± 9.5, BDI = 11.0 ± 6.9). In those with available ECG data, there were also no statistically significant group differences in HTT (mFND: 0.63 ± 0.26; HCs: 0.70 ± 0.21; *p* = .41) or ITPE (mFND: 0.39 ± 1.45; HCs: −0.29 ± 1.43; *p* = .20) scores when controlling for the effect of BMI; BPQ‐awareness scores were also not statistically different (mFND: 112.57 ± 28.11; HCs: 100.38 ± 26.80; *p* = .11). In subgroup analyses, there were also no statistically significant differences in HTT (FND‐seiz = 0.63 ± 0.26, FND‐movt = 0.69 ± 0.17, HC = 0.70 ± 0.21; *p* = .51) or ITPE scores after controlling for the effect of BMI (FND‐seiz = 0.39 ± 1.45, FND‐movt = −0.12 ± 1.47, HC = −0.29 ± 1.43; *p* = .27); BPQ‐awareness scores were also not statistically different (*p* = .26, FND‐seiz = 112.60 ± 28.11, FND‐movt = 104.80 ± 26.50, HC = 100.38 ± 26.79; *p* = .31).

### 3.2 Between‐group VBA, tractography and network lesion mapping findings

**FIGURE 1:**
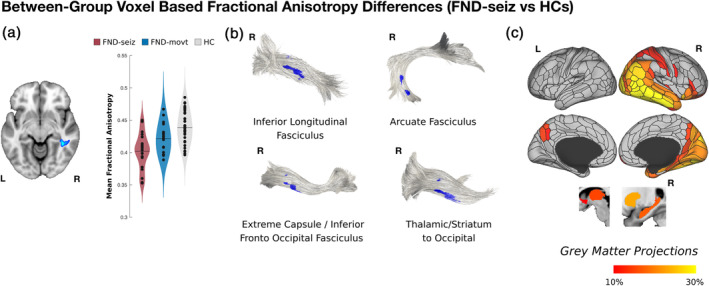
Reduced fractional anisotropy in limbic and associative fiber bundles in patients with functional seizures (FND‐seiz; *n* = 21) compared to healthy controls (HCs; *n* = 38). Panel A shows that voxel‐based analysis (VBA) identified decreased white matter integrity in the right mid‐temporal white matter; this result is adjusted for between‐group age, gender, head motion, depression and trait anxiety scores. Note: in violin plots, data for the functional movement disorders (FND‐movt) subgroup is shown for descriptive purposes only, but all displayed results otherwise pertain to the FND‐seiz vs. HCs comparison. As shown in Panel B, the VBA findings projected onto four right‐lateralized fiber bundles using probabilistic tractography: inferior longitudinal fasciculus, arcuate fasciculus, extreme capsule/inferior fronto‐occipital fasciculus, thalamic/striatum projections to occipital cortex. Panel C identifies the gray matter origins of these white matter alterations in patients with FND‐seiz, predominantly implicating right‐lateralized temporoparietal junction and inferior temporal gyrus areas

Adjusting for covariates, the complete mFND cohort compared to HCs did not show any statistically significant FA differences. In subgroup VBA analyses, patients with FND‐seiz compared to HCs showed reduced right mid‐temporal white matter FA (*t*‐statistic = −5.76; *p*‐value = <.001; Cohen's *d* = 1.56); in tractography analyses, these findings related to four right‐lateralized fiber bundles: inferior longitudinal fasciculus, arcuate fasciculus, extreme capsule/inferior fronto‐occipital fasciculus and thalamic/striatum to occipital cortex projections (see Figure 1). In network lesion mapping, these white matter alterations originated predominantly from right TPJ and inferior temporal gyrus areas.

In analyses comparing FMD‐movt to HCs, there were no statistically significant FA differences adjusting for covariates of non‐interest. There were also no group‐level differences when comparing FA maps between FND‐seiz and FND‐movt subgroups. See Figures S1 and S2 for unadjusted findings.

### 3.3 Relationship between interoceptive accuracy, interoceptive trait prediction error (ITPE) and white matter integrity

**FIGURE 2:**
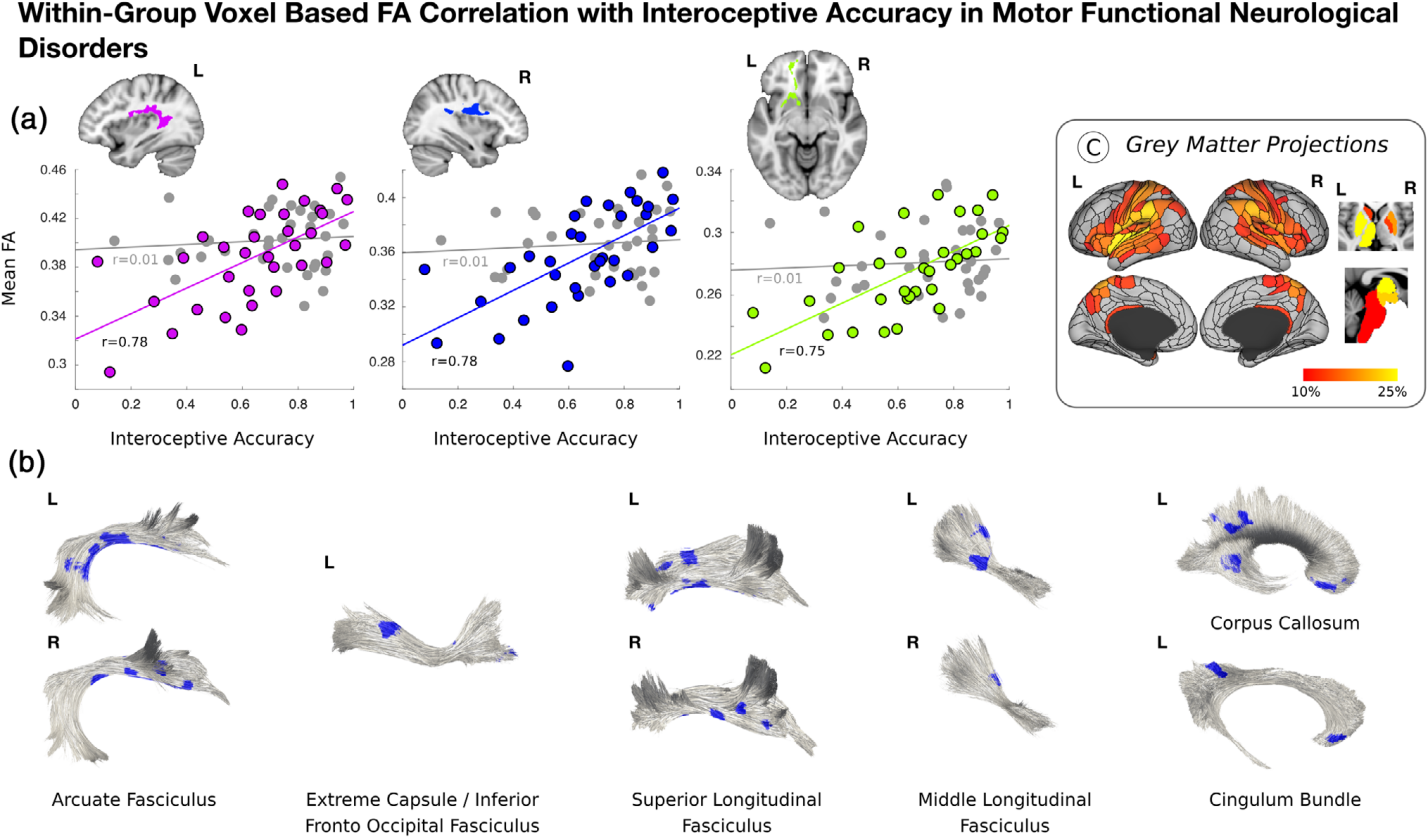
Correlations between fractional anisotropy (FA) and interoceptive accuracy in 33 patients with motor functional neurological disorders (mFND). Panel A shows that across the entire mFND cohort heartbeat counting accuracy positively correlated with bilateral posterior peri‐insular and left ventromedial prefrontal cortex white matter integrity in voxel‐based analyses. For descriptive purposes, scatterplots display the data for mFND patients (colored circles), as well as nonsignificant relationships in healthy controls (gray circles). These within‐group findings were adjusted for age, gender, body mass index, head motion, depression and trait anxiety scores. As shown in Panel B, FA findings related to the following fiber bundles: bilateral arcuate fasciculi, superior/middle longitudinal fasciculi, and left extreme capsules/inferior fronto‐occipital fasciculus, cingulum bundle and corpus callosum. Using network lesion mapping (Panel C), heartbeat counting accuracy correlated robustly with integrity of white matter tracts originating from the bilateral insula, TPJ, putamen and left thalamus among other areas

Across patients with mFND, individual differences in heartbeat counting accuracy positively correlated with bilateral posterior peri‐insular (left: *t*‐statistic = 5.57, *p*‐value = <.001, Cohen's *d* = 0.97; right: *t*‐statistic = 5.67; *p*‐value = <.001; Cohen's *d* = 0.99) and left ventromedial prefrontal (*t*‐statistic = 5.12, *p*‐value = <.001, Cohen's *d* = 0.89) white matter integrity in VBA analyses (see Figure 2 and Figure S3). Only the bilateral posterior peri‐insular VBA findings remained significant adjusting for mFND subtypes and SSRI/SNRI use. Using tractography, FA findings related to the following tracts: bilateral arcuate fasciculi, superior/middle longitudinal fasciculi, and left extreme capsule/inferior fronto‐occipital fasciculus, cingulum bundle and corpus callosum. Using network lesion mapping, heartbeat counting accuracy correlated most robustly with white matter tracts originating from the bilateral insula, TPJ, putamen and left thalamus.

**FIGURE 3:**
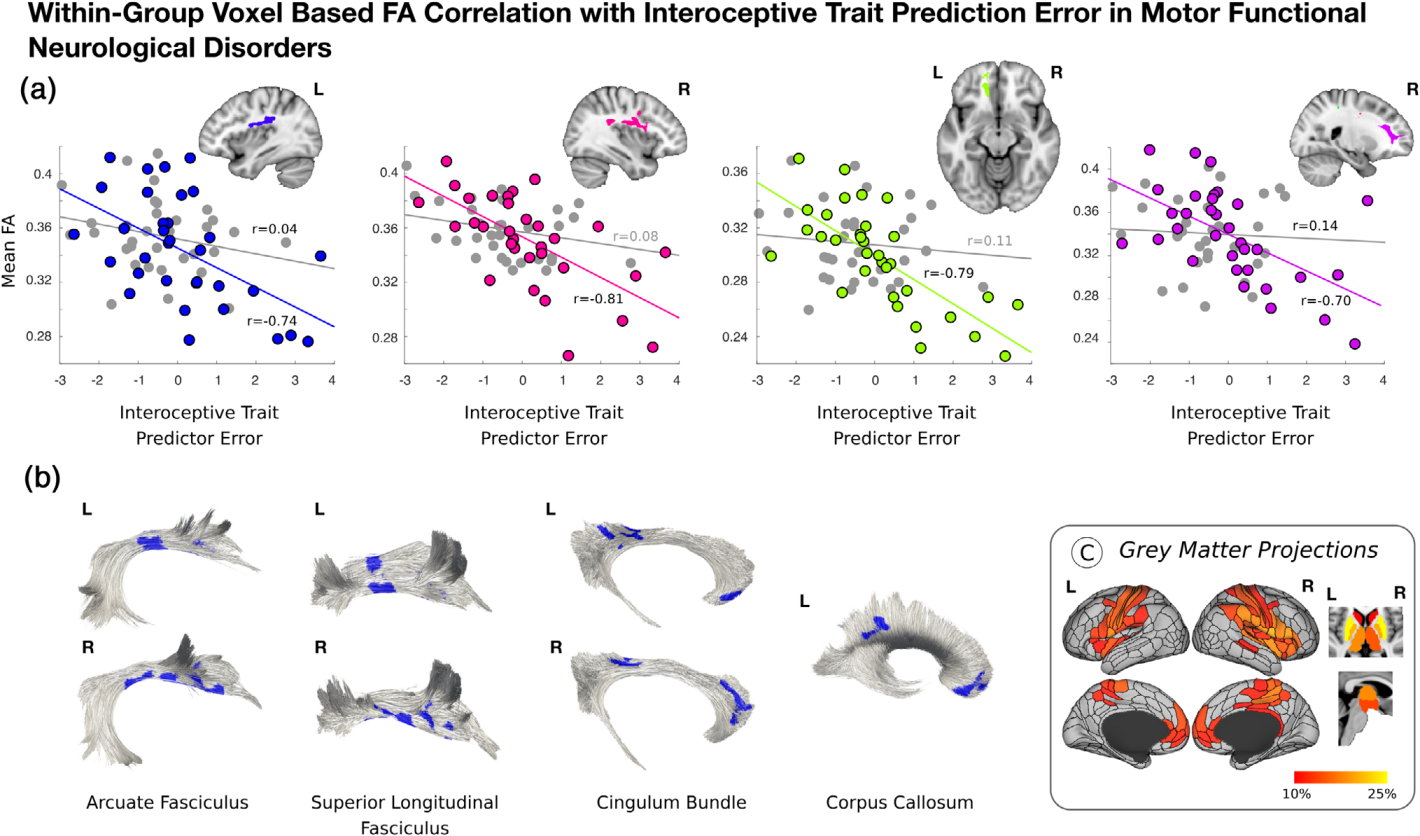
Correlations between fractional anisotropy (FA) and interoceptive trait prediction error in 33 patients with motor functional neurological disorders (mFND). Panel A shows that across the entire mFND cohort interoceptive trait prediction error negatively correlated with bilateral posterior peri‐insular, left ventromedial prefrontal and right dorsal prefrontal white matter integrity in voxel‐based analyses. For descriptive purposes, scatterplots display the data for mFND patients (colored circles), as well as nonsignificant relationships in healthy controls (gray circles). These within‐group findings were adjusted for age, gender, body mass index, head motion, depression and trait anxiety scores. As shown in Panel B, areas of reduced FA related to the following fiber bundles: bilateral arcuate fasciculi, superior longitudinal fasciculi, cingulum bundle, and left corpus callosum. Using network lesion mapping (Panel C), ITPE scores correlated robustly with white matter tracts originating from bilateral insula, ventromedial prefrontal cortices, temporoparietal junction, and putamen among other areas

Across the entire mFND cohort, individual differences in ITPE scores negatively correlated with bilateral posterior peri‐insular (left: *t*‐statistic = −5.17, *p*‐value = <.001, Cohen's *d* = 0.90; right: *t*‐statistic = −5.91, *p* = <.001, Cohen's *d* = 1.03), left ventromedial prefrontal (*t*‐statistic = −5.50, *p*‐value = <.001; Cohen's *d* = 0.96) and right dorsal prefrontal (*t*‐statistic = −4.18; *p* = <.001, Cohen's *d* = 0.73) white matter integrity in VBA analyses (see Figure 3 and Figure S4). These VBA findings remained significant adjusting for mFND subtypes and SSRI/SNRI use. Using tractography, FA findings related to the following tracts: bilateral arcuate fasciculi, superior longitudinal fasciculi, cingulum bundle, and left corpus callosum. Using network lesion mapping, ITPE scores correlated most robustly with white matter tracts originating from the bilateral insula, ventromedial prefrontal cortices, temporoparietal junction, and putamen.

In HCs, there were no statistically significant within‐group relationships between FA, interoceptive accuracy and ITPE scores.

## 4 DISCUSSION

Contrary to study hypotheses, there were no between‐group differences in cardiac interoceptive accuracy and the complete mFND cohort did not show any group‐level FA differences compared to HCs. In subgroup analyses, however, patients with FND‐seiz compared to HCs showed reduced integrity in four right‐lateralized white matter tracts adjusting for depression and trait anxiety: extreme capsule/inferior fronto‐occipital fasciculus, arcuate fasciculus, inferior longitudinal fasciculus, and thalamic/striatum to occipital cortex projections. Network lesion mapping showed that these FND‐seiz related white matter alterations originated in fiber bundles predominantly from right TPJ and inferior temporal gyrus cortices. No statistically significant FA differences were found in FND‐movt compared to HCs or in FND‐seiz compared to FND‐movt. In within‐group mFND analyses, individual differences in interoceptive accuracy positively correlated with microstructural integrity in bilateral posterior peri‐insular and left ventromedial prefrontal white matter, with fiber bundles predominantly originating from the bilateral insula, TPJ, putamen and left thalamus; ITPE scores negatively correlated with white matter alterations originating from similar brain areas—with additional contributions from bilateral ventromedial prefrontal cortices in network lesion mapping analyses. In HCs, individual differences in white matter integrity did not correlate with interoceptive accuracy or ITPE scores—underscoring that biological heterogeneity found in patients with mFND has mechanistic relevance (Perez, Williams, et al., 2017). This is the first neuroimaging study in any FND population investigating brain‐interoception relationships, relating interoceptive abilities to fiber bundles involved in active inference, homeostasis, salience, and multimodal integration (Seeley, 2019; Paulus et al., 2019).

A novel finding is that individual differences in objective interoceptive accuracy and ITPE scores related to white matter integrity in limbic (extreme capsule, cingulum bundle) and associative (arcuate fasciculus, superior longitudinal fasciculus, corpus callosum) tracts in the mFND cohort. Notably, network lesion mapping analyses implicated the insula, TPJ and putamen in these processes. The insula is a primary interoceptive cortex involved in integrating internal feeling states, salience, homeostasis, and self/emotional awareness (Craig, 2009; Paulus & Stein, 2006). In the mFND literature, reduced insula activity correlated with low emotional awareness in an FND‐movt sample (Sojka et al., 2019). Insula structural and functional neuroimaging profiles have also correlated with patient‐reported symptom severity (Diez, Ortiz‐Terán, et al., 2019; Li et al., 2014; Perez, Matin, et al., 2017; Perez, Williams, et al., 2017). Additionally, amygdala link‐step connectivity to the right anterior insula and putamen predicted 6‐month improvement in a mFND cohort (Diez, Ortiz‐Terán, et al., 2019), underscoring the importance of the insula in FND‐related disease and prognostic mechanisms. Furthermore, the putamen is connected to the insula—with studies identifying that insula and putamen co‐activation supports a role for the putamen in modulating motor responses to visceral changes (Napadow et al., 2013; Ruffle et al., 2019). Additionally, it is noteworthy that network lesion mapping implicated bilateral ventromedial prefrontal cortices in ITPE in patients with mFND. ITPE scores measure discrepancies between belief in general interoceptive ability and objective interoceptive performance (Garfinkel et al., 2016); in active inference terms, ITPE can be considered as measuring an overly rigid top‐down belief that fails to be updated using incoming sensory information—thereby making expectations relatively fixed and inaccurate (Paulus et al., 2019). Associations between ITPE and insular and ventromedial prefrontal white matter pathways in our mFND cohort are consistent with a framework that alterations in these brain areas can promote prediction errors, aiding chronic arousal and misperception of bodily symptoms (Paulus & Stein, 2010). Thus, we speculate that associations between individual differences in insula and ventromedial prefrontal cortex related white matter integrity and ITPE scores in patients with mFND reflect an impairment in updating bodily awareness based on lived experiences.

Observations that individual differences in interoceptive accuracy and ITPE scores correlated with TPJ‐related fiber bundles is also consistent with the literature implicating this brain area in the pathophysiology of mFND (Baizabal‐Carvallo et al., 2019). The TPJ, together with cingulo‐insular brain areas, is involved in higher‐order multimodal integration (Sepulcre et al., 2012). Within the context of the neurobiology studies in mFND, right TPJ hypoactivation has been observed during functional tremors compared to volitional movements performed by the same individual (Voon, Gallea, et al., 2010); additionally, task and resting‐state functional connectivity analyses have characterized reduced connectivity between the right TPJ and primary sensorimotor areas, suggesting that impaired feed forward processing may lead to disturbances of self‐agency in mFND (Maurer et al., 2016; Voon, Gallea, et al., 2010; Zito, Wiest, & Aybek, 2020). Studies employing the Libet clock paradigm in FND‐movt and FND‐seiz further support deficits in motor intention awareness (Baek et al., 2017; Jungilligens et al., 2019). Our findings suggest that individual differences in TPJ‐related white matter integrity provide a structural neuroanatomical basis for disturbances in bodily awareness in mFND. More research is needed to investigate the interplay between the TPJ and cingulo‐insular brain areas in the pathophysiology of mFND.

In terms of between‐group neuroimaging findings, FND‐seiz patients showed right‐lateralized corticolimbic white matter pathway disruptions. These findings are notable given higher incidence of right hemispheric structural pathology (Devinsky, Mesad, & Alper, 2001), decreased right‐lateralized cortical thickness (Labate et al., 2012) and rightward uncinate fasciculus asymmetry (Hernando et al., 2015) previously reported in FND‐seiz cohorts. Given the right hemisphere's role in self‐awareness and emotion processing (Devinsky, 2000), we theorize that disruptions in right‐lateralized fiber bundles may promote the dissociation (fragmentation) of affect from visual, auditory and bodily‐perception information. For example, the inferior longitudinal fasciculus is a visual–limbic integration pathway, connecting posterior temporal and occipital cortices to medial temporal structures. Right inferior longitudinal fasciculus lesions can produce a visual‐specific form of derealization (visual hypoemotionality) (Fischer et al., 2016), and depersonalization/derealization scores correlated with right lateral occipital cortical thickness in a mFND cohort (Perez et al., 2018). Additionally, the extreme capsule is a limbic fiber tract connecting the insula with the opercula and medial temporal structures including the amygdaloid complex (Nachtergaele et al., 2019); microstructural alterations within this tract may disrupt bodily awareness (Kleckner et al., 2017). We also speculate that reduced right arcuate fasciculus integrity, a fronto‐insular‐temporal language processing pathway, may reflect emotional‐linguistic deficits (including alexithymia) reported in FND‐seiz patients (Nomi, Schettini, Broce, Dick, & Uddin, 2018).

Regarding the lack of group‐level white matter differences between FND‐movt and HCs, as well as between FND‐movt and FND‐seiz, there are several possibilities to consider. FND‐movt may represent a more heterogeneous mFND subtype relative to FND‐seiz. A similar explanation may apply to the FND‐movt compared to FND‐seiz white matter findings, however a plausible alternative is that white matter differences between FND‐seiz and FND‐movt subtypes may be subtle at best. Moreover, between‐group level findings for the entire mFND cohort compared to HCs prior to adjusting for depression and trait anxiety were similar to the FND‐seiz subgroup vs HCs findings (see Figure S1). This suggests that larger sample size studies are needed to more definitively investigate between‐group differences while controlling for affective comorbidities in mFND. Alternatively, psychiatric controls may help disentangle the neurocircuitry of mFND in relation to its closely associated mood and anxiety comorbidities (Diez et al., 2020; Jenkins et al., 2016). Overall, robust within‐group neuroimaging findings in the context of modest between‐group findings underscore the value of studying individual differences to advance the pathophysiology of mFND, while also highlighting the need for larger sample size studies.

While the primary goal of this study was to investigate white matter—cardiac interoception relationships in patients with mFND, it is important to contextualize the behavioral findings. Previous studies provided evidence for cardiac interoceptive deficits in FND (Koreki et al., 2020; Ricciardi et al., 2014), while the present and two other studies did not find cardiac interoception deficits in mFND (Jungilligens et al., 2019; Pick, Rojas‐Aguiluz, et al., 2020). There may be several explanations for these mixed findings. First, there may be individual differences in cardiac interoception among mFND patients and impaired cardiac interoception may be present only in a subset of mFND individuals. Second, reports about resting cardiac activity may be based on prior knowledge of heart rate and on other non‐interoceptive factors so the ability to count heartbeats may not be a valid indicator of interoceptive accuracy (Ring & Brener, 2018). Furthermore, only a third of non‐clinical populations demonstrate accurate heartbeat detection suggesting prominent heterogeneity across individuals (Khalsa & Lapidus, 2016). Future imaging and non‐imaging interoceptive studies should also test interoceptive perturbations outside of the individuals locus of control to more comprehensively assess interoception in mFND (Paulus et al., 2019).

Study limitations include modest sample size, psychiatric comorbidities, psychotropic medication use, and phenotypic heterogeneity. We adopted a transdiagnostic approach across FND‐seiz and FND‐movt given arguments by several groups that these populations are on a clinical continuum (Mula, 2013; Perez et al., 2015), however, we acknowledge that this remains debated in the field (Kanaan, Duncan, Goldstein, Jankovic, & Cavanna, 2017). We did not perform a structured psychiatric interview limiting description of categorical psychiatric comorbidities. This study also did not include a patient‐reported symptom severity or health‐related quality of life measure (e.g., Short Form Health Survey‐36 [Pick, Anderson, et al., 2020]), which prevented testing of associations between interoceptive accuracy, ITPE scores and disease severity. Interoceptive accuracy and ITPE analyses could be limited by floor effects. Moreover, we used ECG to measure heartbeats (instead of finger pulse plethysmograph) and the signal loss associated with R‐peak extraction may have contributed to the lack of difference in cardiac interoceptive accuracy. While our neuroimaging analyses accounted for SSRI/SNRI use which is a strength, other psychotropic medications may have confounded results—underscoring the importance of larger sample size cohorts to adequately allow for a broader set of post‐hoc analyses. In terms of DTI methodology, limitations in accounting for cross fibers require additional clarification. The future acquisition of serial DTI data is also needed to verify measurement reliability, including to replicate our network lesion mapping findings (Shahim, Holleran, Kim, & Brody, 2017). Lastly, more work is needed to clarify roles for the dorsal prefrontal cortex, basal ganglia, thalamus and their white matter connections in interoceptive processing in mFND.

In conclusion, this DTI study identified several right‐lateralized limbic and associative tracts in the pathophysiology of FND‐seiz and investigated brain‐interoception relationships across the spectrum of mFND. Individual differences in interoceptive accuracy and trait prediction error mapped onto microstructural alterations in fiber bundles originating from the insula and TPJ, advancing our understanding of the pathophysiology of mFND.

## CONFLICT OF INTEREST

D. L. P. has received honoraria for continuing medical education lectures on functional neurological disorder.

## Supporting information


**Supplementary Figure S1** Adjusting only for age, gender and head motion, reduced fractional anisotropy in limbic and associative fiber bundles in patients with motor functional neurological disorders (mFND; *n* = 38) compared to healthy controls (HCs; *n* = 38) was identified. Displays voxel‐based analysis and probabilistic tractography findings.

**Supplementary Figure S2** Adjusting only for age, gender, and head motion, displays voxel based analysis and probabilistic tractography findings comparing patients with functional seizures (FND‐seiz; *n* = 21; Panels A‐B) or functional movement disorders (FND‐movt; *n* = 17; Panel C) to healthy controls (HCs; *n* = 38).

**Supplementary Figure S3** Correlations between interoceptive accuracy and individual differences in white matter integrity in patients with motor functional neurological disorders (*n* = 33). Panel A shows voxel‐based analysis, probabilistic tractography and network lesion mapping findings adjusting for only age, gender, body mass index (BMI), and head motion. Panel B shows the same correlations adjusting for age, gender, BMI, head motion, depression, trait anxiety and FND subtypes; Panel C shows the findings adjusting for age, gender, BMI, head motion, depression, trait anxiety and antidepressant use.

**Supplementary Figure S4** Correlations between interoceptive trait prediction error and individual differences in white matter integrity in patients with motor functional neurological disorders (*n* = 33). Panel A shows voxel‐based analysis, probabilistic tractography and network lesion mapping findings adjusting for only age, gender, body mass index (BMI), and head motion. Panel B shows the same correlations adjusting for age, gender, BMI, head motion, depression, trait anxiety and FND subtypes; Panel C shows the findings adjusting for age, gender, BMI, head motion, depression, trait anxiety and antidepressant use.

**Appendix** **S1**: Supporting information

## Data Availability

Anonymized data and the neuroimaging scripts will be shared with qualified researchers on request to the corresponding author following approval by the local ethics committee.
