# Supplementary figures and images for: Individual differences in interoceptive accuracy and prediction error in motor functional neurological disorders: A DTI study
Source: Hum Brain Mapp. 2020 Dec 7;42(5):1434–45. doi: 10.1002/hbm.25304 (PMC7927304; doi:10.1002/hbm.25304)

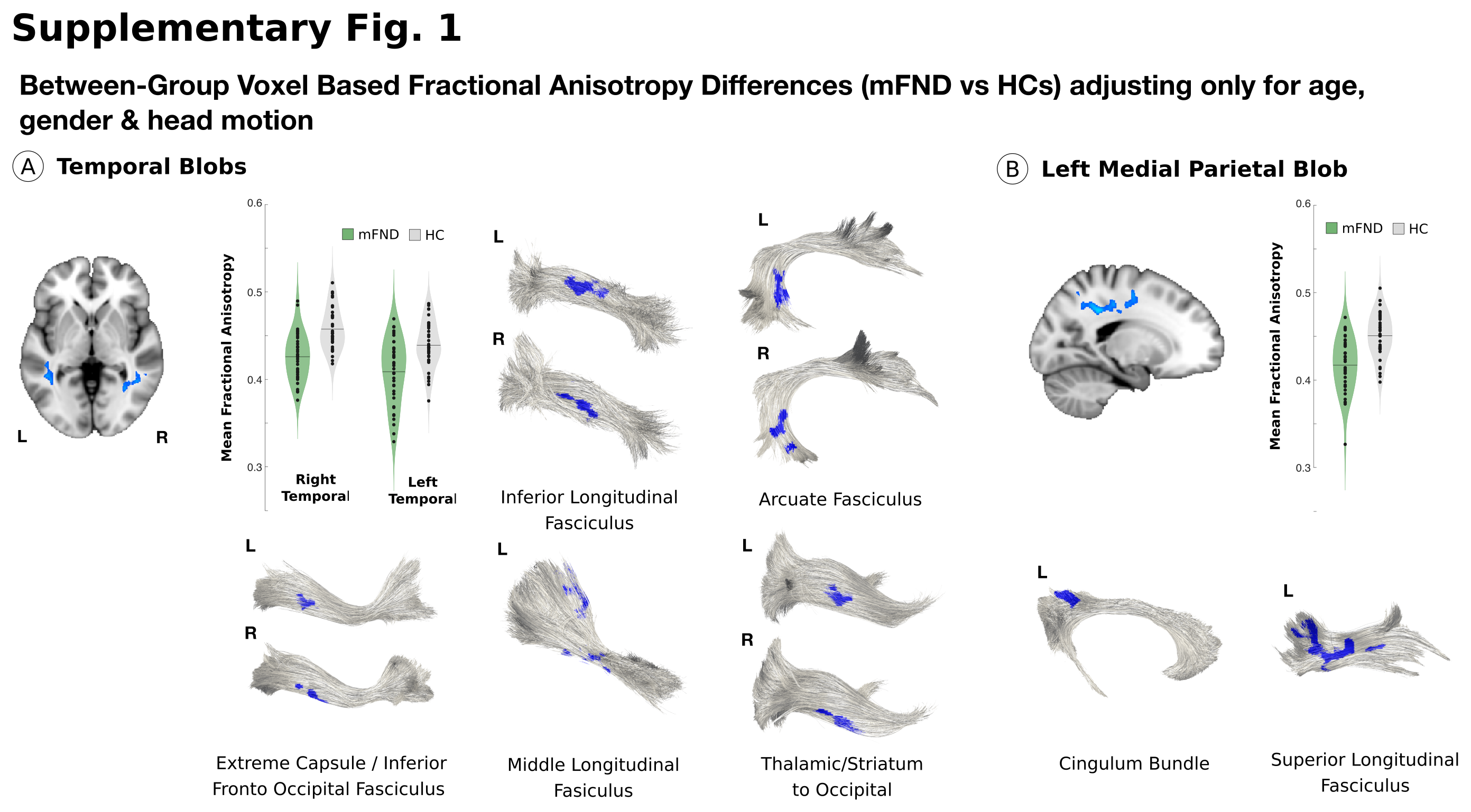

Supplement: Supplementary file 1 — Supplementary Figure S1 Adjusting only for age, gender and head motion, reduced fractional anisotropy in limbic and associative fiber bundles in patients with motor functional neurological disorders (mFND; n = 38) compared to healthy controls (HCs; n = 38) was identified. Displays voxel‐based analysis and probabilistic tractography findings. [file HBM-42-1434-s001.tiff]

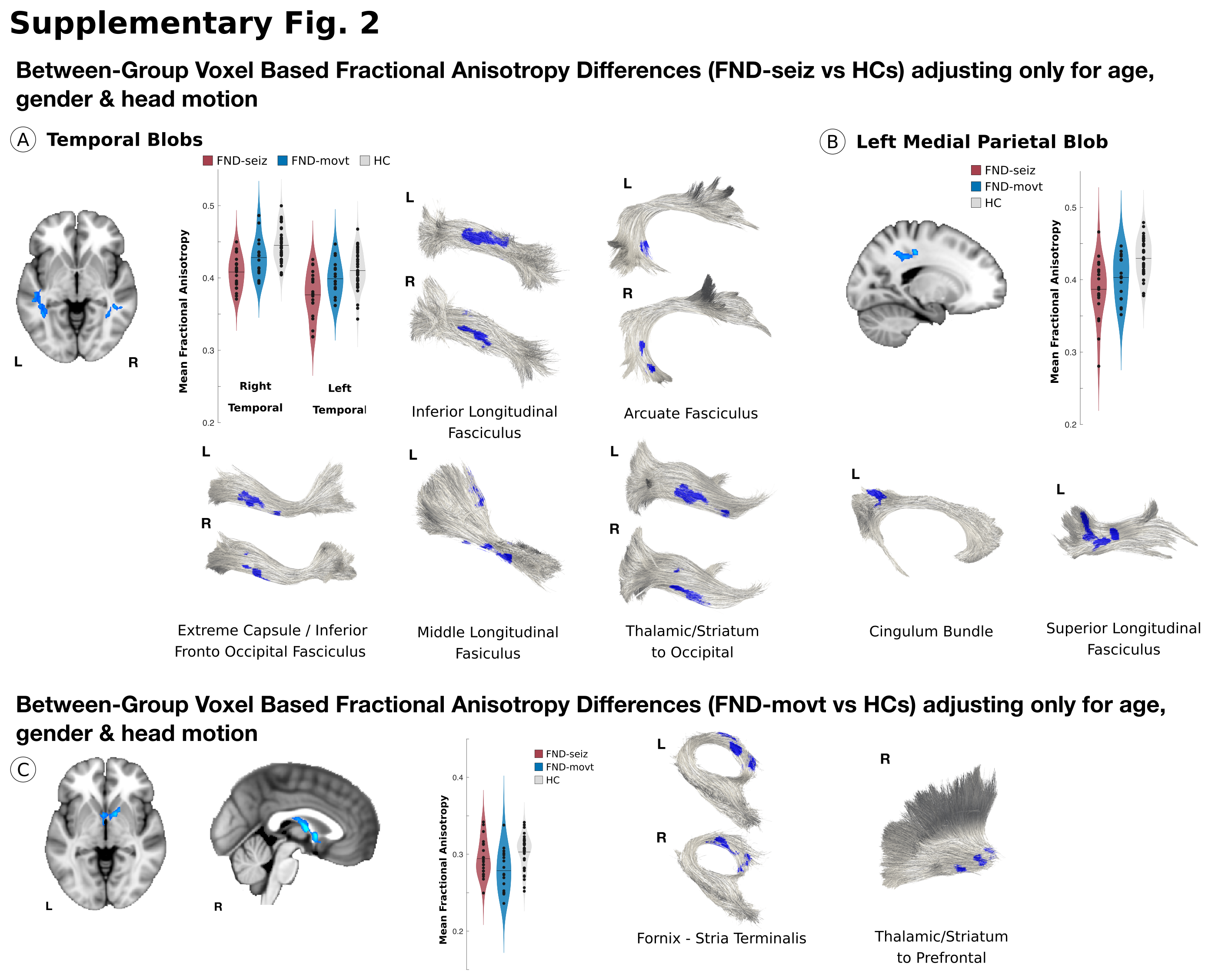

Supplement: Supplementary file 2 — Supplementary Figure S2 Adjusting only for age, gender, and head motion, displays voxel based analysis and probabilistic tractography findings comparing patients with functional seizures (FND‐seiz; n = 21; Panels A‐B) or functional movement disorders (FND‐movt; n = 17; Panel C) to healthy controls (HCs; n = 38). [file HBM-42-1434-s002.tiff]

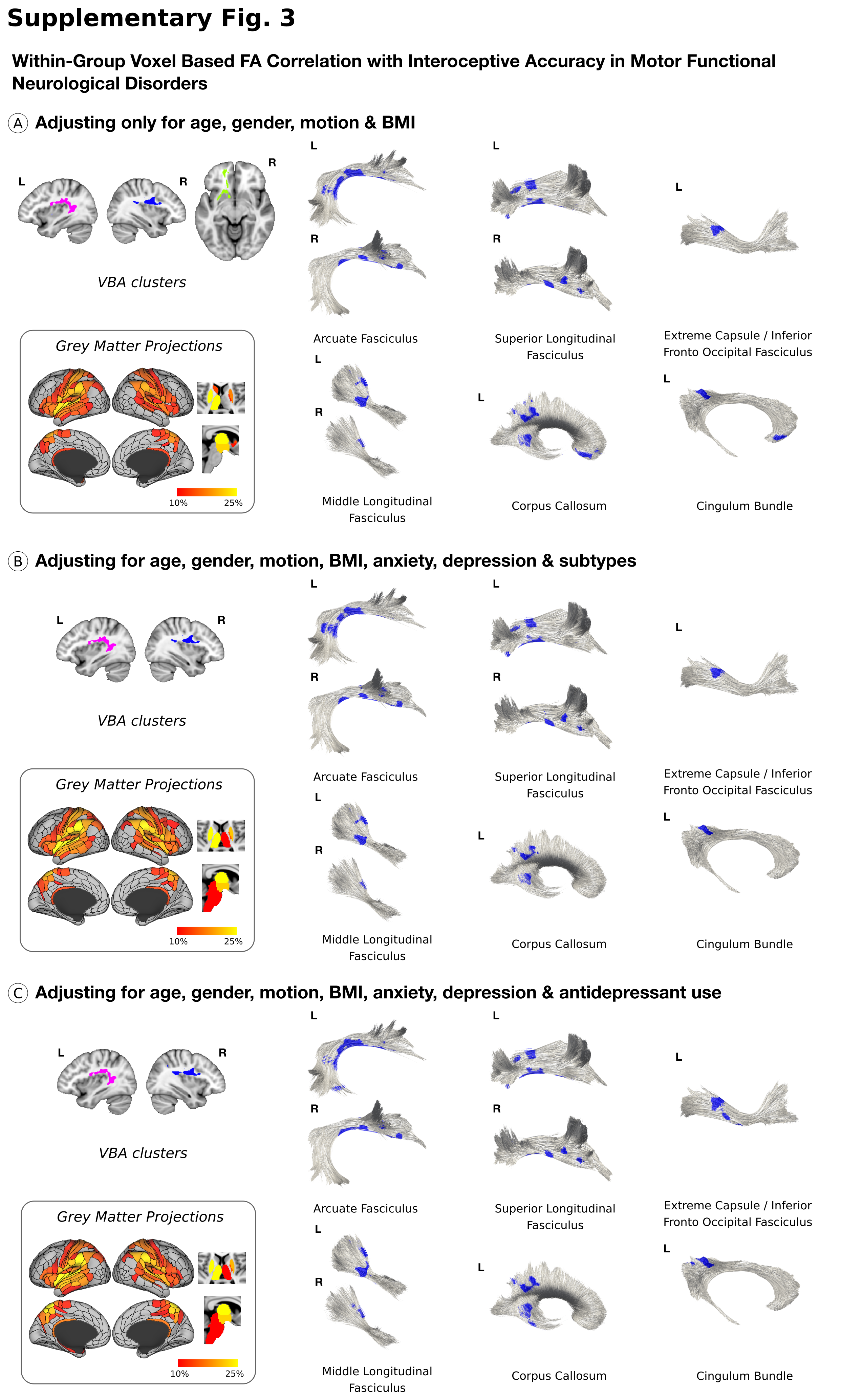

Supplement: Supplementary file 3 — Supplementary Figure S3 Correlations between interoceptive accuracy and individual differences in white matter integrity in patients with motor functional neurological disorders (n = 33). Panel A shows voxel‐based analysis, probabilistic tractography and network lesion mapping findings adjusting for only age, gender, body mass index (BMI), and head motion. Panel B shows the same correlations adjusting for age, gender, BMI, head motion, depression, trait anxiety and FND subtypes; Panel C shows the findings adjusting for age, gender, BMI, head motion, depression, trait anxiety and antidepressant use. [file HBM-42-1434-s003.tiff]

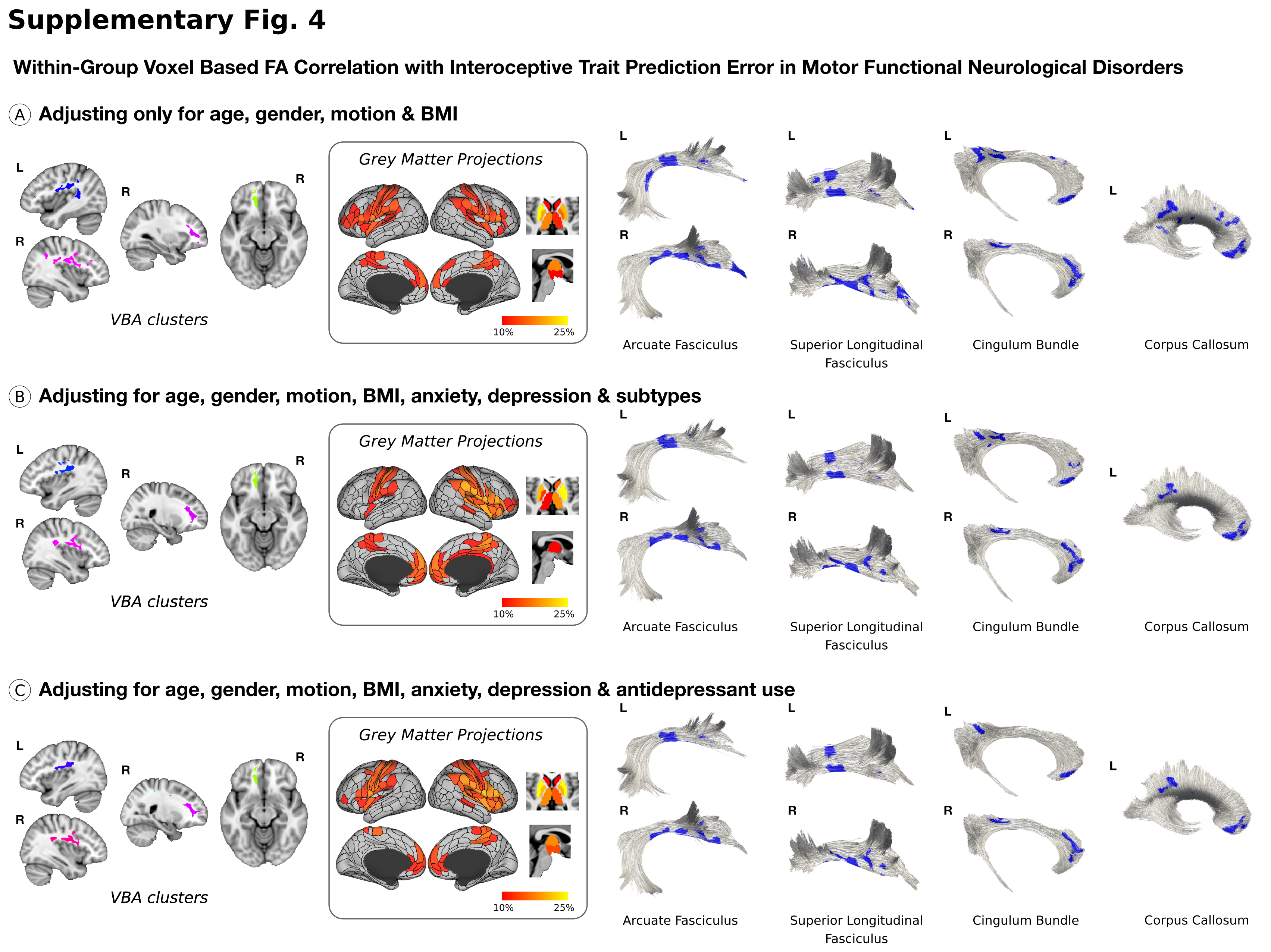

Supplement: Supplementary file 4 — Supplementary Figure S4 Correlations between interoceptive trait prediction error and individual differences in white matter integrity in patients with motor functional neurological disorders (n = 33). Panel A shows voxel‐based analysis, probabilistic tractography and network lesion mapping findings adjusting for only age, gender, body mass index (BMI), and head motion. Panel B shows the same correlations adjusting for age, gender, BMI, head motion, depression, trait anxiety and FND subtypes; Panel C shows the findings adjusting for age, gender, BMI, head motion, depression, trait anxiety and antidepressant use. [file HBM-42-1434-s004.tiff]
