# Supplementary material for: Individual differences in interoceptive accuracy and prediction error in motor functional neurological disorders: A DTI study
Source: Hum Brain Mapp. 2020 Dec 7;42(5):1434–45. doi: 10.1002/hbm.25304 (PMC7927304; doi:10.1002/hbm.25304)
Supplement: Supplementary file 5 — Appendix S1: Supporting information [file HBM-42-1434-s005.docx]

**Supplementary Methods**

***MRI acquisition***

Scans were obtained using a 3T Siemens Magnetom Prisma. High resolution 3D T1-weighted magnetization prepared rapid gradient-echo (MPRAGE) sequences were acquired with the following parameters: 1mm isotropic voxels; 240 sagittal slices; acquisition matrix size=252×252; repetition time (TR)=2300 ms; echo time (TE)=2.33 ms; field of view (FOV)=224mm. Fluid-attenuated inversion recovery (FLAIR) sequences were also obtained with the following parameters: 1mm isotropic voxels; 192 sagittal slices; acquisition matrix size=256×256; TR=6000 ms; TE=387 ms; FOV=256mm. Multishell diffusion-weighted images were acquired using pulsed gradient-spin-echo echo-planar-imaging (EPI) under the following parameters: 2mm isotropic voxels; 72 axial slices; TR = 9000ms; TE = 82ms; acquisition matrix size=128×128; FOV=256mm; with 4 diffusion-weighted shells at b=200 s/mm2 (3 volumes), b=500 s/mm2 (6 volumes), b=1000 s/mm2 (30 volumes), b=2500 s/mm2 (30 volumes) and 6 b=0 s/mm2 scans interspersed throughout.

***MRI preprocessing***

The T1-weighted data was preprocessed using *FreeSurfer* 6.0.0 (<http://surfer.nmr.mgh.harvard.edu/>) cortical parcellation and volumetric segmentation. This pipeline included: removal of non-brain tissue using a hybrid watershed/surface deformation procedure; automated Talairach transformation; segmentation of the subcortical white matter and deep grey matter structures; intensity normalization; delineation of the grey matter-white matter boundary; automated topology correction; and surface deformation following intensity gradients to optimally identify the grey/white and grey/cerebrospinal fluid boundaries. Surface based registration projected the Desikan parcellation to individual subjects. The FLAIR image was used to improve the pial surface delineation.

Diffusion-weighted image preprocessing performed using FMRIB Software Library v5.0.7 (FSL) included: eddy current correction; gradient vector rotation to compensate for head motion; local fitting of the diffusion tensor at each voxel; and computation of FA maps. The total head motion index was computed as a covariate of non-interest to account for motion confounds.^31^ Non-linear transformation between each subject’s FA map space and T1-weighted image was computed and concatenated to the non-linear transformation between the subject’s T1-weighted image and MNI152 standard template. This procedure transformed each subject’s FA map to MNI152 standard space.
